# Hydrophobically Associating Polymers Dissolved in Seawater for Enhanced Oil Recovery of Bohai Offshore Oilfields

**DOI:** 10.3390/molecules27092744

**Published:** 2022-04-24

**Authors:** Fei Yi, Bo Huang, Chengsheng Wang, Xiaoxu Tang, Xiujun Wang, Quangang Liu, Yanhui Su, Shijia Chen, Xiaoyan Wu, Bin Chen, Jing Zhang, Dianguo Wu, Shuai Yu, Yujun Feng, Xin Su

**Affiliations:** 1 State Key Laboratory of Offshore Oilfield Exploitation, Tianjin 300452, China; yifei@cnooc.com.cn (F.Y.); huangbo@cenertech.com.cn (B.H.); wangchsh2@cenertech.com.cn (C.W.); wangxj89@cnooc.com.cn (X.W.); liuqg@cenertech.com.cn (Q.L.); suyh@cnooc.com.cn (Y.S.); chenshj2@cenertech.com.cn (S.C.); wuxy7@cnooc.com.cn (X.W.); chenbin8@cnooc.com.cn (B.C.); ex_zhangj16@cnooc.com.cn (J.Z.); 2 CNOOC EnerTech-Drilling and Production Co., Tianjin 300452, China; 3 CNOOC Research Institute, Beijing 100027, China; 4 Tianjin Branch of CNOOC Ltd., Tianjin 300451, China; tangxx@cnooc.com.cn; 5 Polymer Research Institute, State Key Laboratory of Polymer Materials Engineering, Sichuan University, Chengdu 610065, China; wu-dianguo@foxmail.com (D.W.); yushuai@stu.scu.edu.cn (S.Y.); yjfeng@scu.edu.cn (Y.F.)

**Keywords:** hydrophobically associating polymers, sea water, viscosity, enhanced oil recovery, polymerization

## Abstract

As compared to China’s overall oil reserves, the reserve share of offshore oilfields is rather significant. However, offshore oilfield circumstances for enhanced oil recovery (EOR) include not just severe temperatures and salinity, but also restricted space on offshore platforms. This harsh oil production environment requires polymers with relatively strong salt resistance, solubility, thickening ability, rapid, superior injection capabilities, and anti-shearing ability. As a result, research into polymers with high viscosity and quick solubility is recognized as critical to meeting the criteria of polymer flooding in offshore oil reservoirs. For the above purposes, a novel hydrophobically associating polymer (HAP) was prepared to be used for polymer flooding of Bohai offshore oilfields. The synthetic procedure was free radical polymerization in aqueous solutions starting at 0 °C, using acrylamide (AM), acrylic acid (AA), 2-acrylamido-2-methylpropane sulfonic acid (AMPS), and poly(ethylene glycol) octadecyl methacrylate (POM) as comonomers. It was discovered that under ideal conditions, the molecular weight of HAP exceeds 2.1 × 10^7^ g⋅mol^−1^. In a simulated reservoir environment, HAP has substantially greater solubility, thickening property, and salt resistance than conventional polyacrylamide (HPAM), with equivalent molecular weight. Finally, the injectivity and propagation of the two polymers in porous media were investigated. Compared with HPAM, which has a similar molecular weight, HAP solution with the concentration of 0.175% had a much better oil displacement effect in the porous medium, which can enhance oil recovery by 8.8%. These discoveries have the potential to pave the way for chemical EOR in offshore oilfields.

## 1. Introduction

Petroleum is a sort of strategic resource that is important to a country’s politics, economy, and military. It is seen as an essential component affecting the country’s progress. Petroleum availability is also related to socioeconomic advancement [1,2]. In recent years, China’s total petroleum consumption has considerably outstripped its total supply, and increasing oil recovery is an effective approach to ensure China’s petroleum supply. Accordingly, improving the oil and gas recovery in each oilfield is significant to China’s energy strategy. Among these, the priority is to increase the recovery efficiency of offshore oilfields producing heavy crude oil. It reveals that China’s proportion of heavy oil to offshore oil is greater than 70% [3]. The ratio of heavy oil production to overall crude oil production has surpassed 60% [4]. Several limitations, however, hinder the recovery of waterflooding to between 18% and 20%, including high viscosity of crude oil, low productivity, high exploration costs, restricted area of offshore platform, and limited load-bearing capacity [5,6]. Even when the entire well pattern is encrypted, the waterflooding recovery may be as low as 25% [6], significantly less than the typical waterflooding recovery in China’s mainland heavy oilfields. Therefore, it is important to increase the recovery of crude oil in offshore heavy oilfields.

Enhanced oil recovery (EOR) is a technology for increasing crude oil recovery [7,8]. As a technique for EOR, chemical flooding entails injecting chemicals (such as a water-soluble polymer) into the injection water to improve the interaction of the fluid and crude oil and so increase the swept volume, thereby enhancing crude oil recovery. Polymer flooding, as the most extensively utilized approach of chemical flooding, offers the advantages of simplicity of operation, pronounced effects, and low cost [9,10]. Consequently, polymer flooding was determined to be the most likely application and most promising strategy for enhancing recovery of crude oil [11].

Partially hydrolyzed polyacrylamide (HPAM) is mostly used for polymer flooding at onshore oilfields [11], but it is incompatible with offshore oilfields. From a standpoint of molecular structure, HPAM is ill-suited for application in conditions with high temperature and high salt. Electrostatic repulsion within and between HPAM molecules generated by carboxylic groups has the potential to significantly thicken fresh water. However, in saline water or seawater, inorganic cations can mask the electrostatic repulsion, leading to polymer chain fracture and a decrease in hydrodynamic radius, all of which contribute to the macroscopic reduction of viscosity [12]. The viscosity of the aqueous solution of HPAM decreases with increasing temperature. At 75 °C, the amide group can be hydrolyzed to form carboxylic groups (−COOH). Divalent cations in saline water further reduce viscosity of aqueous solution; due to the presence of multivalent cations, complexation between −COOH and Ca^2+^ or Mg^2+^ may occur, resulting in phase separation [13]. In such cases, the viscosity of the HPAM solution reduces significantly, making oil recovery of offshore oilfield less than ideal. Furthermore, from a practical standpoint, the load-bearing capacity and platform space of offshore oilfields are smaller than those of onshore oilfields. These characteristics hampered the widespread application of polymer flooding technology in offshore oilfields, especially using HPAM. Therefore, novel polymers for EOR should exhibit reasonable salt tolerance, rapid solubility, and high viscosity, as well as outstanding injection properties and anti-shearing ability.

There are numerous strategies to overcome thermal and salt intolerance of HPAM, including increasing its molecular weight [14,15], generating hydrophobically associating polymers [16,17,18], and introducing comonomers with thermal and salinity tolerance [19,20,21]. HPAM with a high molecular weight has a comparatively large hydrodynamic radius, resulting in relatively high initial and residual viscosities, which induces increased temperature thermal and salt resistance [14]. A hydrophobically associating polymer is a water-soluble polymer that contains a trace number of hydrophobic groups on the hydrophilic macromolecular chains of HPAM [16]. The hydrophobic groups’ associating behavior in an aqueous solution generates aggregation between macromolecule chains, generating micro-network structure and raising the hydrodynamic radius, which has a comparatively strong effect on increasing viscosity [17]. Introducing thermal-resistant and salt-tolerant comonomer can improve the thermal and salt stability of the main chain of HPAM, because the introduced monomers, such as *N*-vinylamine and 2-acrylamido-2-methylpropane sulfonic acid (AMPS) [22], cannot react with ions and can inhibit the hydrolysis of amide groups.

Essentially, a polymer employed in offshore oilfields must have not only a good capacity of increasing viscosity, but also instant solubility, due to the environment of high temperature and high salinity of offshore oilfields, as well as the limited space of offshore platforms. But normal HPAM is not suited for offshore oilfields, because seawater has high salinity and contains divalent cations. Therefore, a novel hydrophobically associating polymer (HAP) was prepared to be used for polymer flooding of Bohai offshore oilfields. The aqueous solution of HAP with low concentration was created using simulated Bohai seawater [23]; Table 1 shows the component of Bohai seawater, whose salinity is 3.26 × 10^5^ mg/L; compared with normal HPAM, the HAP solution demonstrates a thickening effect and instant solubility when employed at the same temperature (75 °C) and salinity as Bohai seawater.

## 2. Results and Discussion

### 2.1. Optimization of Polymerization

**Scheme 1:**
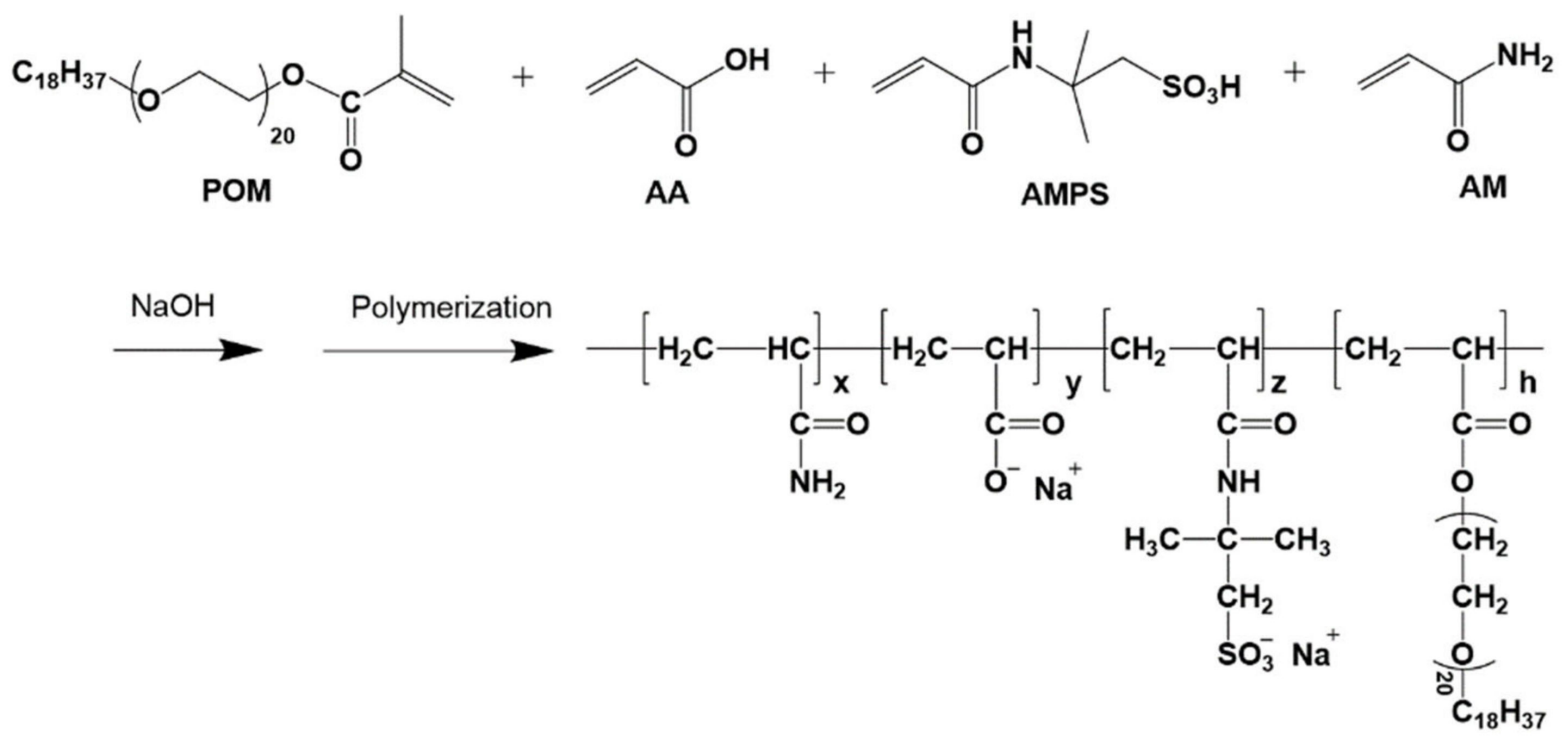
The polymerization reaction for HAP used in the work.

The monomers for synthesizing HAP polymer in the research are AM, AA, AMPS and poly(ethylene glycol) octadecyl methacrylate (POM). Among them, AMPS is a kind of monomer with thermal and salt resistance; POM is a hydrophobic monomer that also contains a long poly(ethylene oxide) segment, which contributes significantly to instant solubility of HAP. Low-temperature aqueous solution polymerization [24] was chosen as the method of choice for the synthesis of HAP with high molecular weight, high viscosity, and resistance to high temperature and salinity (Scheme 1).

Several factors, including monomer ratio, pH value, reaction time and reaction temperature, can affect the copolymerization behavior of all monomers in an aqueous solution during polymer synthesis; so, the polymerization was completed as 52 runs (Tables S1–S3). Bohai seawater (Table 1) was selected as solvent, and the resultant polymer was utilized to make saline solutions with concentration of polymer as 0.175%; the solution viscosity and dissolution time at 75 °C were used as indications for inquiry. The appropriate monomer ratio and reaction condition are determined by the effect of the polymer generated via the chosen polymerization system on high viscosity and short dissolution time (Tables S1–S3). The influencing factors for the polymerization reaction of HAP on the solution viscosity and dissolution time, from Tables S1–S3, are represented in Figure S1, including content of AMPS and POM, pH value of reactant solution, temperature before polymerization, and duration time of polymerization.

It is helpful to incorporate AMPS groups into the HAP polymer for enhancing thermal and salt resistance. The reason is that sulfonic acid side groups on HAP from AMPS are ionized in the solution and induce electrostatic repulsion, stretching the polymer molecular chain of HAP in the aqueous solution and increasing the apparent viscosity. From Figure S1a, it is found that with the increase of AMPS content, the dissolution rate of the polymer enhanced, while the viscosity also slightly increased; but when AMPS continued to increase from 16% to 20%, the growth of viscosity is no longer very obvious. Moreover, the massive addition of AMPS is not helpful for synthesis of a high-molecular-weight polymer. The controlled addition of AMPS 16% is appropriate.

The introduction of POM monomer also increases apparent viscosity caused by the hydrophobic associative effect. Figure S1b indicates that as POM comonomer increases from 0.5% to 3.0%, the dissolution time of the polymer increases from 25 min to 125 min, while the viscosity increases from 9.8 mPa⋅s to 99.1 mPa⋅s. Therefore, the introduction of POM are two sides to the same coin, meaning POM can increase the viscosity of the polymer solution and make the polymer become difficult to dissolve at the same time. When POM was added as 2.0%, the viscosity of the polymer solution was about 55.0 mPa⋅s, which is proper for polymer flooding.

As shown in Figure S1c, as the pH of the reactant solution for polymerization increases from 2.0 to 12.0, the produced polymer dissolves more rapidly, but the viscosity of the polymer solution steadily decreases. The reason is as follows: When polymerization starts under acidic conditions, cross-linking between polymer molecular chains occurs easily, resulting in insolubility of the obtained polymer; when polymerization is performed under strongly alkaline conditions, the ester group in POM monomer is easy to be hydrolyzed, resulting in a decrease in polymer viscosity. As a result, the ideal pH for polymerization is approximately 8.0.

The data about polymerization time are summarized in Figure S1d. Polymerization time varies from 3 to 15 h; the polymer dissolution time becomes longer, but the viscosity steadily increases. Short reaction time, namely less than 10 h, results in fast dissolution speed and lower viscosity of the polymer solution, because the produced polymer has low molecular weight; when the reaction time is extended to more than 12 h, the reaction solution become elastically gel-like and the obtained polymer has high molecular weight, which lengthens dissolution time and improves high viscosity. Thus, in order to complete the polymerization process and create a polymer with a high molecular weight, the polymerization period remains as at least 12 h.

From Figure S1e, as the temperature at polymerization increases from 0 to 40 °C, the polymer dissolution rate becomes faster, but the viscosity of the produced polymer solution gradually decreases. When polymerization occurs at low temperature (0 °C), the auto-acceleration of polymerization rate is not obvious. As initiation proceeds, the temperature gradually increases and the molecular weight slowly increases, so the obtained polymer has high molecular weight, causing the polymer solution to remain viscous; however, as the original temperature gradually increases, it intensifies auto-acceleration of polymerization, decreasing the molecular weight of the polymer and lowering the viscosity of the polymer solution. Therefore, the staring temperature of polymerization is chosen as 0 °C.

By optimizing and screening the conditions of polymerization reaction, the HAP of No. 46 run in Table S3 was chosen as the target polymer for further study after extensive consideration and comparison, due to its rapid dissolution ability (dissolution time, 32 min;) and high viscosity (55.8 mPa⋅s); the properties of the chosen HAP and commercial HPAM are shown in Table 2.

**Figure 1:**
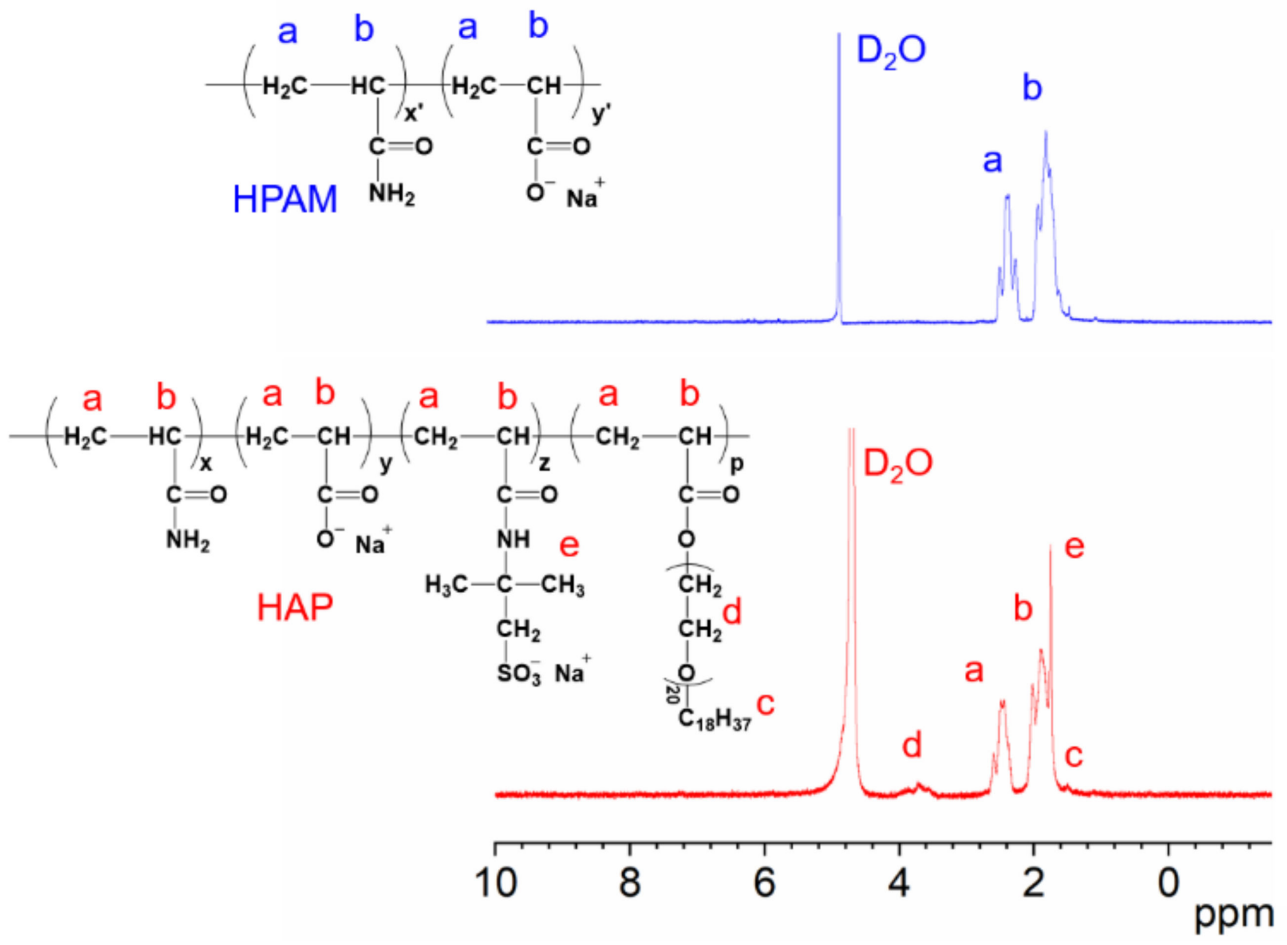
The comparison between ^1^H NMR spectra of HAP and HPAM, where (**a**–**e**) denote the representative hydrogens in HPAM and HAP polymers, respectively, and their corresponding peaks in ^1^H NMR spectra.

The ^1^H NMR analysis was performed to determine whether HAP under optimal reaction condition, from No. 46 run, was synthesized as expected, compared with the commercial HPAM. Figure 1 demonstrates that the chemical changes at 1.6–2.0 ppm and 2.2–2.5 ppm correspond to the main chain of the polymer’s proton absorption peaks. The chemical shift at 1.8 ppm corresponds to the proton absorption peaks of the methyl groups from AMPS in the HAP polymer. The chemical shift peak of the methylene group (−CH_2_OCH_2_−) from coupled to oxygen on the functional monomer POM in the polymer is 3.7–4.9 ppm. The hydrogen chemical shift in long-chain alkyl group on the side chains of POM are located at 1.8–2.0 ppm. These results demonstrated that AMPS and POM had successfully polymerized on the molecular main chain of HAP.

**Figure 2:**
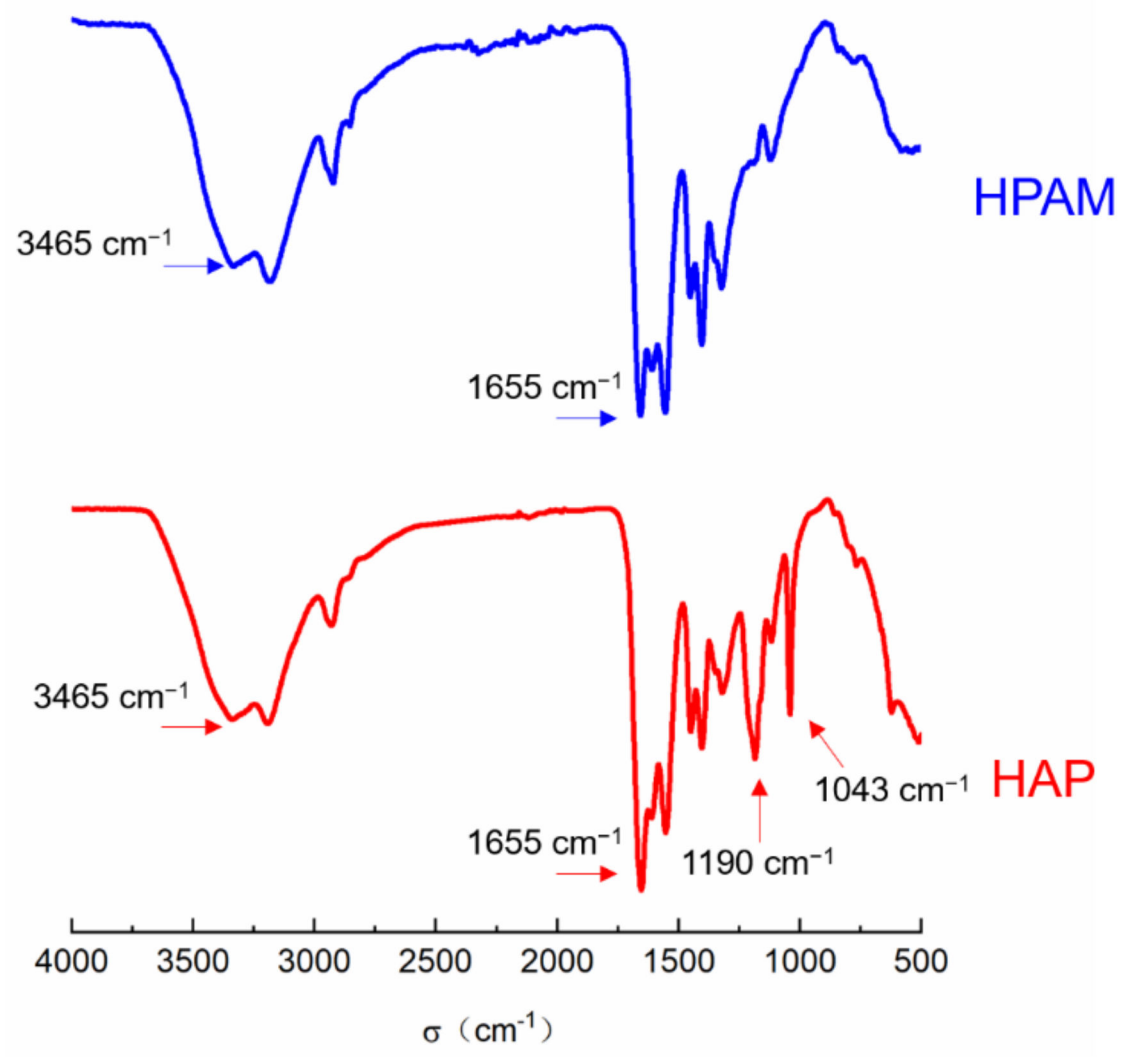
The comparison between FTIR spectra of HAP and HPAM.

The functional groups in polymers also can be analyzed from FTIR spectra (Figure 2). The two largest absorption peaks in the spectra at 1655 cm^−1^ and 3465 cm^−1^ are the N-H stretching vibration of the amide group and the stretching vibration of the carbonyl group C=O, respectively, indicating that the main components of the functional polymer come from AM and AA. The absorption peaks at 1190 cm^−1^ are the absorption peaks of the sulfonic acid group, showing the presence of the AMPS component in HAP. The absorption peak at 1043 cm^−1^ is the C-O stretching vibration of ether groups, proving the presence of the POM functional monomer in the HAP polymer.

### 2.2. Solid Content of Polymer

Due to their high hydrophilicity, HPAM and its copolymer are prone to absorb moisture. According to the requirement of actual use, dried polymer powder should have a solid content of greater than 90%. The testing indicates that the solid content of HAP and HPAM was 92% and 91%, respectively, which was greater than 90% (Table 2). This indicates that these samples are highly susceptible to absorbing moisture from the air. The precise solid content offers support for designing the ensuing experiments for polymer solutions.

### 2.3. Molecular Weight of Polymer

**Figure 3:**
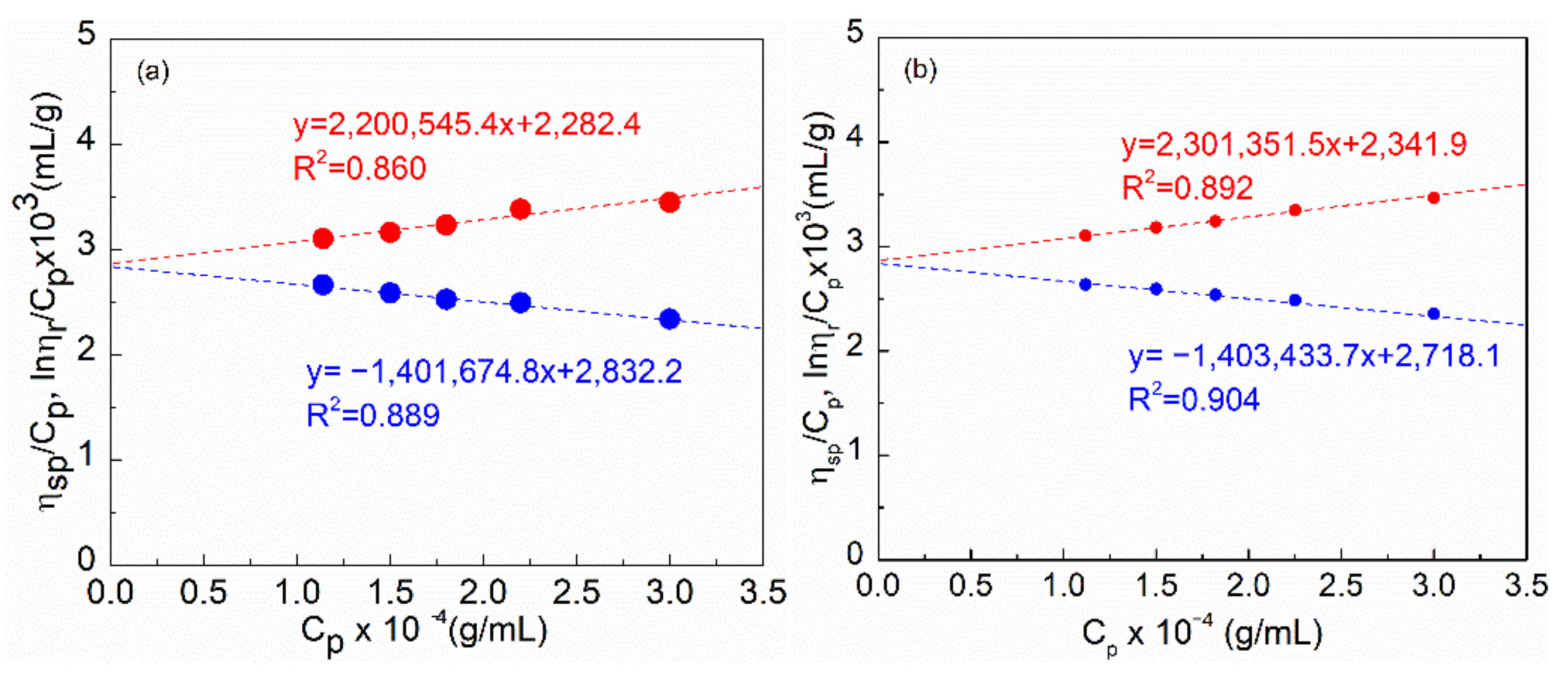
η_sp_/C_p_ and lnη_r_/C_p_ plotted as a function of polymer concentration for (**a**) HAP and (**b**) HPAM at 30 °C.

As seen in Figure 3, the Huggins equation [25] used for the polymer solution of HAP and HPAM showed outstanding linear fitting results with the R^2^ (coefficient of determination) greater than 0.85. This demonstrates that this approach was capable of determining the typical viscosity of HAP and HPAM. When concentration was low, the molecule chain was comparatively stretched in solution, resulting in a low K_H_ value. The molecular weight was calculated to be 2.1 × 10^7^ and 2.2 × 10^7^, based on intrinsic viscosity. As can be observed, HAP has a similar molecular weight as HPAM. It should be emphasized that Feng et al. completed the corresponding research [26], using the capillary viscometer with the Huggins equation to measure the molecular weight of the 10 million level of HPAM, and it is proved that the method is applicable.

### 2.4. Solubility of Polymer

**Figure 4:**
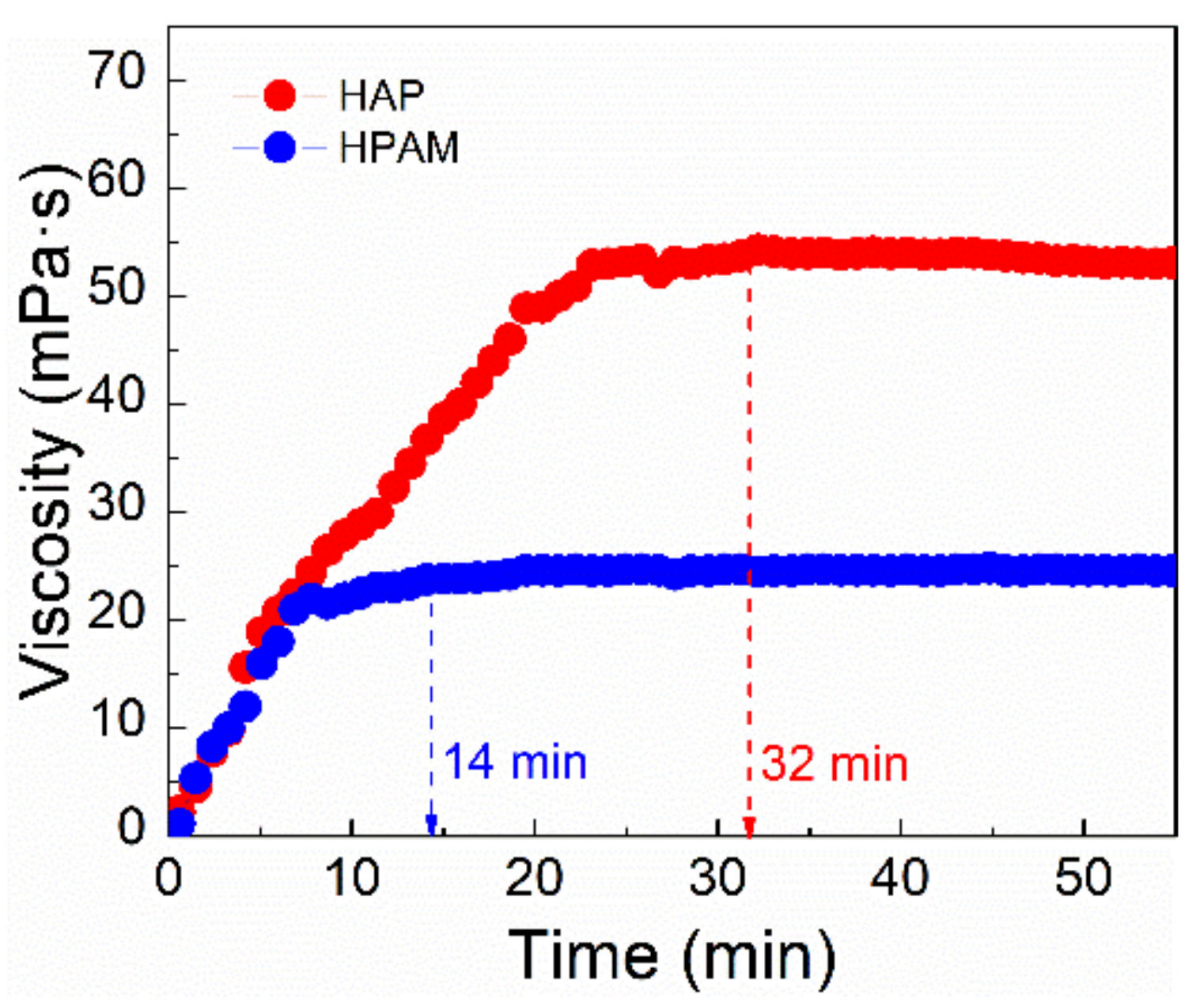
The apparent viscosity variation of HAP and HPAM solutions with dissolving time in Bohai seawater at 75 °C (Particle size of polymer powder, 40–120 mesh; polymer concentration, 0.175%; shear rate, 7.34 s^−1^).

The solubility of the polymer is meaningful in determining whether it can be utilized for polymer flooding. A polymer with a particularly lengthy dissolution time would result in a significant cost loss for applications in EOR [27]. Two steps in the microscopic view may be identified in the dissolving of dry polymer powder. To begin, water molecules infiltrated the polymer, causing the dry polymer to transform into a swelled gel form. The polymer’s molecular chain then spread into the water from the swelled gel. The solubilities of HAP and HPAM polymers were compared to the same quantity in stimulated saline in this paper. Figure 4 illustrates how apparent viscosity varies over time.

The growing trend of the apparent viscosity of HAP after 32 min remained steady, indicating that the HAP polymer has good solubility in Bohai seawater. This might be due to the addition of a SO_3_^−^ hydrophilic group to HAP, which shortened the time required for solution in brine. Also, the polyoxyethylene ether groups of POM monomer is hydrophilic, which can help dissolve HAP in aqueous solution. In contrast, the maximum viscosity of the HPAM solution was attained in 14 min, indicating that the HAP polymer was substantially more soluble in brine; but the maximum of HPAM is 26.3 mPa⋅s, much lower than the solution of HAP.

### 2.5. Thickening Ability of Polymer Solution

**Figure 5:**
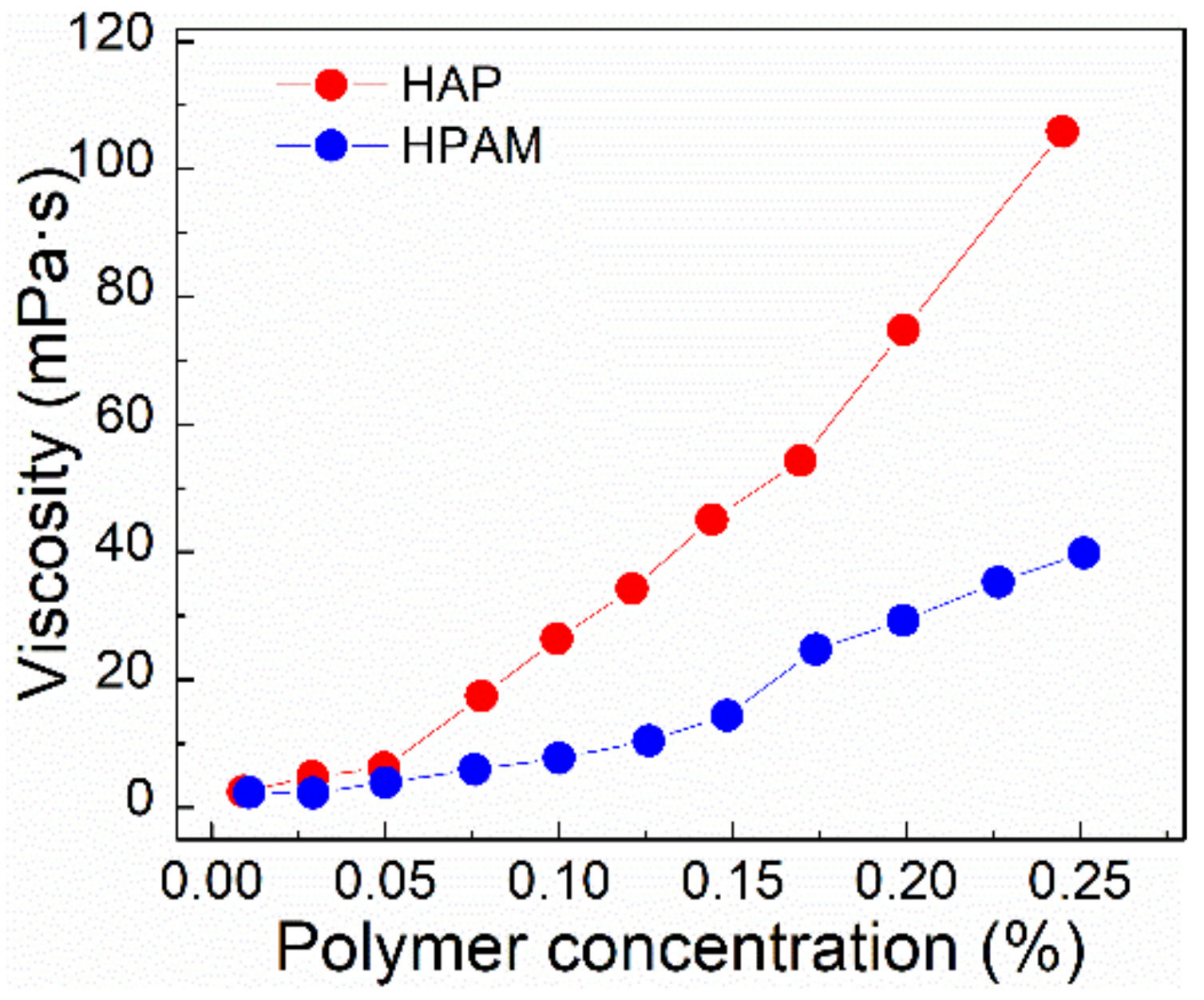
For HAP and HPAM, the viscosity as a function of concentration is displayed at 75 °C. (Particle size of polymer powder, 40–120 mesh; salinity, 3.26 × 10^5^ mg/L; shear rate, 7.34 s^−1^).

The polymer’s thickening property is critical for EOR. The optimal polymer for EOR should be able to be employed at low concentrations while maintaining a high viscosity, resulting in economic benefits. It was necessary to plot the apparent viscosity of both polymer solutions as a function of polymer concentration in order to compare their thickening abilities. As shown in Figure 5, the viscosity of the HAP solution is somewhat greater than that of the HPAM solution throughout the concentration range, and this difference becomes more pronounced when the polymer concentration exceeds 0.05%. For example, at a polymer content of 0.175%, the HAP solution has a viscosity of 55.8 mPa⋅s, which is much higher than the HPAM solution (26.3 mPa⋅s). The increased viscosity of the HAP solution is due to the addition of the salt-tolerant comonomer AMPS and hydrophobic associating monomer POM to the molecule chain of HAP. As indicated previously, the structure of AMPS and POM renders HAP stable and insensitive to the ions present in Bohai seawater; hence, HAP polymer, which contains AMPS and POM as comonomers, is more stable and has a greater viscosity in Bohai seawater.

The optimal viscosity of the displacing fluid is a compromise between a favorable mobility ratio, i.e., a higher recovery efficiency, and a cost-effective concentration. According to the research from Li et al. [28], the optimal viscosity of a polymer solution in EOR is dependent on the viscosity ratio of the polymer solution to crude oil (η_p_/η_o_), with a higher η_p_/η_o_ resulting in a better recovery efficiency. They found η_p_/η_o_ as 0.7 is appropriate when the viscosity of crude oil is in the range between 40 mPa⋅s and 90 mPa⋅s. The viscosity of crude oil from Bohai oilfields is 78 mPa⋅s at 75 °C, and the concentration of the polymer solution was selected as 0.175%, whose viscosity is 55.8 mPa⋅s.

### 2.6. Shear Tolerance of Polymer Solutions

Mechanical deterioration of the polymer occurs inevitably during the dissolution, dispersion, and pumping of the dry polymer powder in the actual process of polymer flooding in oilfields. The viscosity variations of polymer solutions at shear rate (7.34–100 s^−1^) at 75 °C were measured to verify the suitability of HAP polymers for shearing processes of polymer flooding.

**Figure 6:**
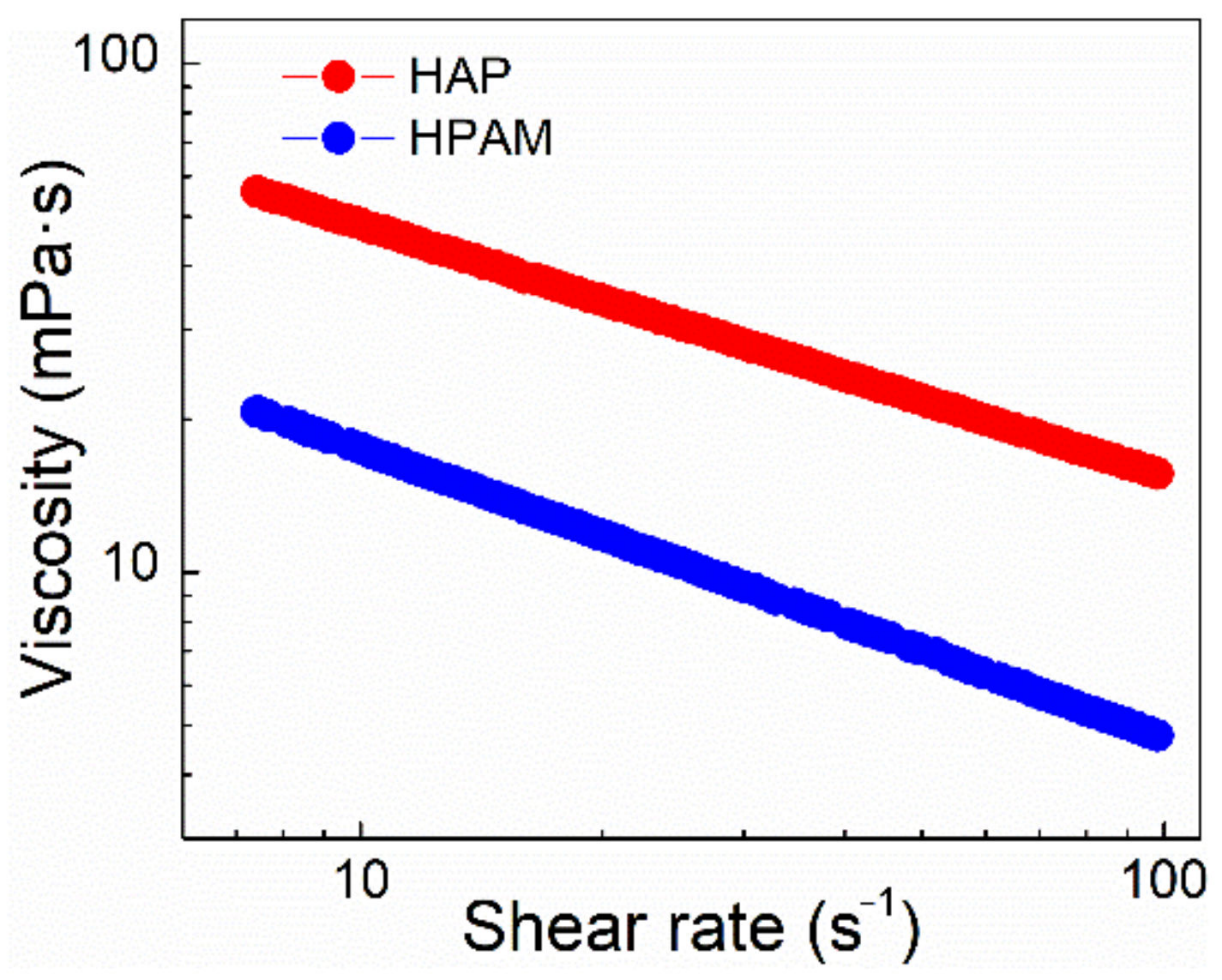
The effect of shear rate on apparent viscosity of polymer solutions at 75 °C. (Polymer concentration, 0.175%; salinity, 3.26 × 10^5^ mg/L).

The connection between apparent viscosity and shear rate for HAP and HPAM solutions is depicted in Figure 6. The apparent viscosity decreased with the shearing rate, which is consistent with the fact that the viscosity of normal polymer solutions reduces with shearing [29]. When the shearing rate was increased from 7.34 s^−1^ to 100 s^−1^, the viscosity retention of the HAP and HPAM polymer solutions was 27.8% and 22.8%, respectively. It shows that the HAP solution has a better shear resistance than the HPAM solution. The reason is that the smaller absolute value of the slope means the decrease of the apparent viscosity of the polymer solution was slower with the increasing shearing rate, and the shear resistance was better. The shear resistance of HAP was significantly better than that of HPAM, owing to the associative effect of hydrophobic chains of HAP. In comparison, the HPAM structure would be further destroyed by shearing. The addition of a functional monomer POM with a hydrophobic associative chain may produce the physical network among molecular chains of HAP, hence increasing the shear resistance of HAP.

### 2.7. Salt Tolerance of Polymer Solutions

Salt is present in all types of water used to create polymer solutions, including oilfield formation water and sewage. The electrostatic shielding action of salt on carboxyl groups in the polymer and the hydration removal impact may both contribute to a reduction in the viscosity of the polymer solution. The simulated Bohai seawater was diluted by pure water, and the brine with different salinity was obtained. The viscosity of the HAP solutions with different salinity was tested and compared with those of HPAM to see if the two types of polymers have relatively better salt resistance.

**Figure 7:**
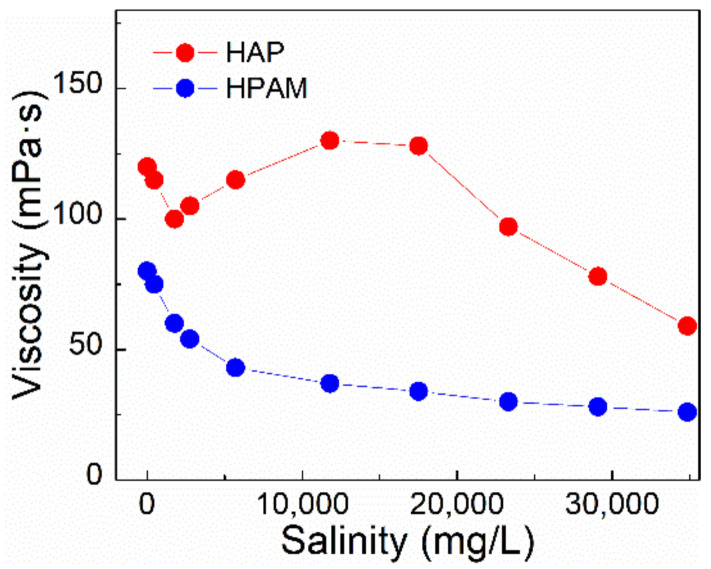
The effect of salinity on apparent viscosity of polymer solutions at 75 °C. (Polymer concentration, 0.175%; shear rate, 7.34 s^−1^).

The viscosity changes of HAP and HPAM solutions can be seen in Figure 7 with different salinity. In comparison to HPAM, the trend of HAP’s viscosity with increasing salinity revealed significant variances that may be classified into three categories. When the concentration of brine was less than 2000 mg/L, the viscosity decreased rapidly with increasing concentration. However, the viscosity of the HAP solution increased from 95 to 139 mPa⋅s at salinity between 2000 and 12,000 mg/L, indicating a clear salt thickening phenomenon attributed to the hydrophobic association [6], showing the property of salt resistance. Following that, the size of polymer chains was reduced with the help of an electrostatic shield, and the salt thickening phenomenon was eliminated when salinity was increased even further. The AMPS unit in the molecular structure of HAP decreased the hydration removal effect of salt ions to the polymer chain. Moreover, the non-ionic groups in the HAP polymer showed electroneutrality and insensitivity to salt, making the curling degree of the molecule chain lower than the normal polymer and improving its salt resistance. Therefore, the increased range of the viscosity of the HAP solution was relatively smaller with saline concentration. Additionally, Figure S1 shows the pH of all the polymer solutions is higher than that of brine with different salinity, indicating that the polymer itself carries the sodium carboxylate, resulting in enhancement of the pH value.

### 2.8. Long-Term Thermal Stability of Polymer Solutions

Because the migration of a polymer solution slug from injectors to producers might take more than half a year, long-term thermal stability is a significant quality for a polymer that will be used in EOR. In the operation of the transportation process, the polymer solution is constantly in touch with the high-temperature environment of the oil reservoir. For real use of polymer solutions in EOR, it is required to first determine the long-term thermal stability of polymer solutions.

**Figure 8:**
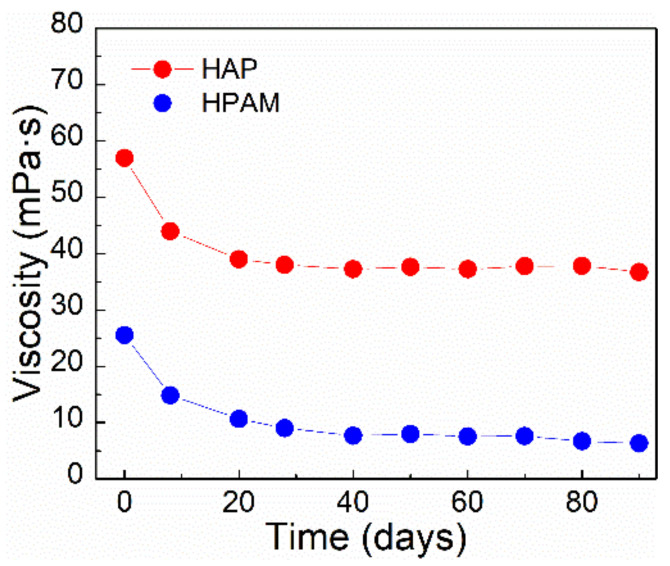
The long-term thermal stability of polymer solutions based on apparent viscosity over time at 75 °C. (Polymer concentration, 0.175%; salinity, 3.26 × 10^5^ mg/L; shear rate, 7.34 s^−1^).

As seen in Figure 8, the HAP solution exhibits high thermal stability when compared to the HPAM solution. After 90 days of ageing at 75 °C, the viscosity of the HAP solution is still more than 30 mPa⋅s, which is 20 mPa⋅s greater than the viscosity of the HPAM solution. The reason for HAP’s viscosity retention is that HAP contains the more thermally stable comonomer AMPS and the hydrophobic associative monomer POM. As previously stated, AMPS’s structure efficiently prevents the amide group from being hydrolyzed. Thermal stability of HAP contributes to higher viscosity retention over the period of the long-term oil displacement process in reservoir environments.

### 2.9. Core Displacement Experiment

Resistance factor (RF) and residual resistant factor (RRF) were used to describe the ability to decrease the water–oil mobility ratio and core permeability by the injection of the polymer solution, respectively. Generally, a larger value of RF means larger seepage resistance of the polymer solution in the core pore, which is beneficial to the enlargement of the swept volume. A larger value of RRF means a larger decrease range of core permeability, which is beneficial to improving crude oil recovery [24]. Table S4 shows the basic parameters of the artificial cores used in this work and Table S5 shows the RF and RRF values after injecting the polymer solution into the core. It demonstrates that at the cores with the same permeability, both the RF and RRF values of HAP were higher than those of HPAM with the same concentration (0.175%), because the viscosity of the HAP solution was 35 mPa⋅s higher than the HPAM solution. The increase of the viscosity of the polymer solution could enlarge the displaced swept volume, and more core pores were available to the polymer solution, leading to the increment of the RRF value.

**Figure 9:**
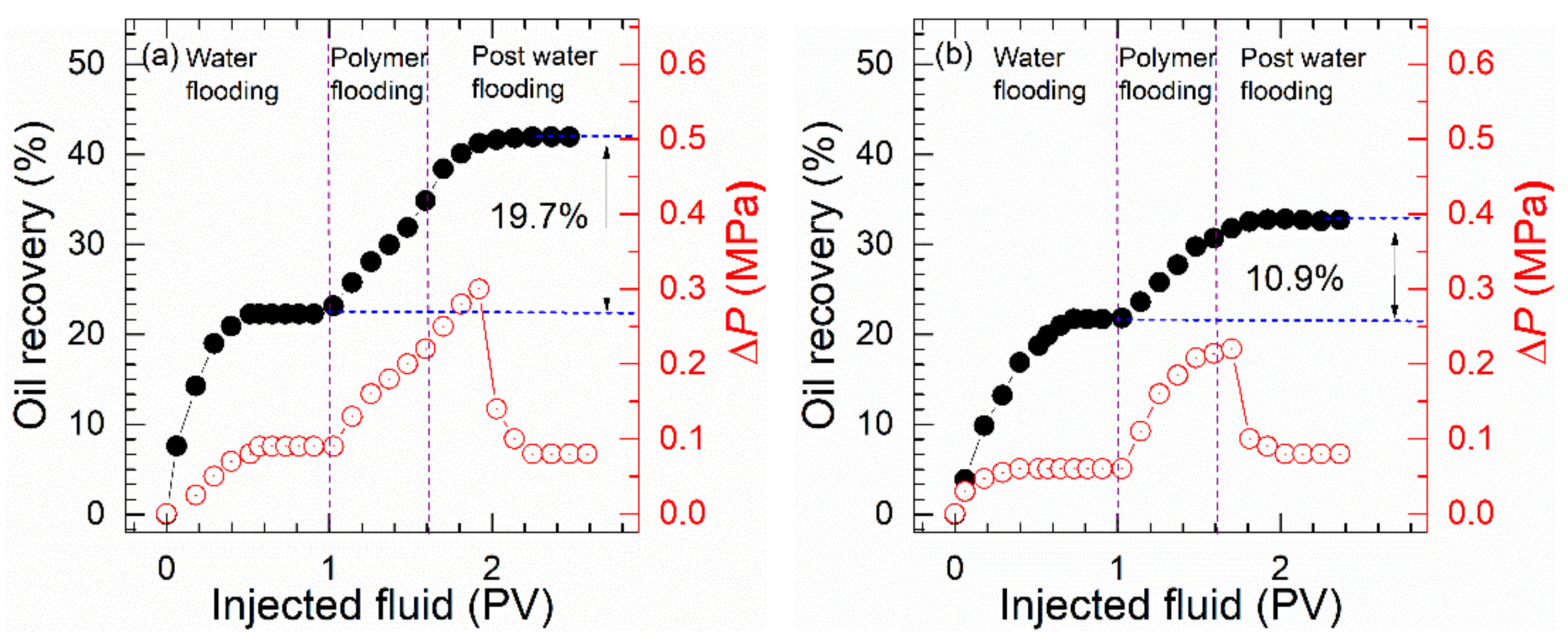
Oil recovery factor and flooding pressure plotted as a function of injected volume for solution of (**a**) HAP and (**b**) HPAM. (Polymer concentration, 0.175%; salinity, 3.26 × 10^5^ mg/L; injection rate of 1.0 mL/min).

The curves in Figure 9 show the changes in the oil recovery efficiency and pressure with injection volumes of HAP and HPAM solutions at 75 °C. The corresponding recovery parameters can be seen in Table S6. The pressure changes after the injection of 0.3 PV polymer should be paid special attention. The peak pressure of the HPAM solution was 0.23 MPa, and that of the HAP solution was 0.31 MPa. This is related to the higher viscosity and viscoelasticity of HAP solution in saline, which increased the retention amount of polymer in porous core media and got a higher-pressure value. Therefore, the oil displacement efficiency of the HAP solution would be higher, realizing a larger scavenging area and 8.8% more oil recovery than HPAM. This indicates that at the same polymer concentration, HAP has a better economic value and a better application prospect than HPAM in polymer flooding for Bohai reservoirs.

## 3. Experimental Procedures

### 3.1. Materials

Acrylamide (AM), acrylic acid (AA) 2-acrylamido-2-methylpropanesulphonic acid (AMPS), ammonium per-sulfate [(NH_4_)_2_S_2_O_8_], sodium hydrogen sulfite (NaHSO_3_), and 2,2-Azobis [2-(2-imidazolin-2-yl) propane] dihydrochloride (VA-044) were purchased from Sigma—Aldrich (Burlington, MA, USA). Poly(ethylene glycol) octadecyl methacrylate (POM) was supplied from Zhangjiagang Render Chemicals Co., Ltd. (Suzhou, China). Polyacrylamide (HPAM) was bought from Adamas-Beta (Shanghai, China). Sodium hydroxide (NaOH), ethanol, and sodium chloride (NaCl) were obtained from Chron Chemicals (Chengdu, China). The deionized water (Resistivity, 18.25 M·cm) was produced using a CDUPT-III ultrapure water purification system (Chengdu Ultrapure Technology Co., Ltd., Chengdu, China). The heavy oil and cores were supplied by CNOOC EnerTech-Drilling and Production Co., Tianjin, China.

### 3.2. Polymerization

The following is a typical HAP preparation procedure that utilizes free radical polymerization starting at 0 °C. AM (184 g), AA (70 g), AMPS (60 g), and POM (7 g) were dissolved in 400 g deionized water at 0 °C and the pH adjusted to 8.0 using NaOH. The solution was then transferred to a thermostatic container and refrigerated to 0 °C using a constant nitrogen flow (30.0 mL/min). Following that, the solution was gradually supplemented with the initiators VA-044 (0.01 g), NaHSO_3_ (0.01 g), and (NH_4_)_2_S_2_O_8_ (0.01 g) dissolved in deionized water. To ensure a thorough reaction, the polymerization was kept at 12 h. Following polymerization, the polymer was dissolved in deionized water for approximately 5 h to achieve full dissolution. The completed product was first dried at 40 °C, then dissolved in deionized water and repeatedly precipitated with excess ethanol before being freeze-dried to obtain the final white powder. Scheme 1 illustrates the chemical route.

### 3.3. Molecular Structure Characterization

The polymer sample was dissolved in D_2_O to get a solution with the concentration of 20 mg/L and was transferred to an NMR tube. The ^1^H NMR spectra were obtained by an NMR spectrometer (ASCEND 800 MHz, Bruker Co., Karlsruhe, Germany).

Infrared absorption spectra were obtained by Fourier infrared spectrometer (TENSOR 27, Bruker Co., Karlsruhe, Germany) after compression of polymer samples with KBr.

### 3.4. Measurement of Viscosity

All rheological measurements were carried out using a Physical MCR 302 rotating rheometer with concentric cylinder shape CC27 (Anton Paar, Graz, Austria). The test temperature was set using a Peltier temperature control device. All samples were kept stable at test temperature for 5 min prior to data collection in all experiments.

### 3.5. Dissolution Time

The dry powder of the polymer was dissolved in simulated Bohai seawater (salinity, 3.26 × 10^5^ mg/L) and was stirred at the speed of 400 r/min in a water bath at 75 °C. The viscosity changes with time were tested. The dissolution time was measured when 95% of the final viscosity was reached.

### 3.6. Measurement of Molecular Weight

At 30 °C, a 1.0 M NaCl aqueous solution and five polymer aqueous solutions of varying concentrations were placed in an IVS400 automatic capillary viscometer (Zonwon Technology Co., Ltd., Hangzhou, China) with a 0.55 mm capillary inner diameter. The running times of polymer solutions (t_1_) and solvents (t_0_) were utilized to determine the relative viscosity η_r_ = t_1_/t_0_ and the specific viscosity η_sp_ = (t_1_ − t_0_)/t_0_. The intrinsic viscosity, [η], was determined by extrapolating to infinite dilution and calculating the intercept using Huggins and Kraemer diagrams. From [η], one can get the viscosity-average molecular weight of the polymer using the Mark–Houwink equation (Equation (1)):

[η] = kM_η_^α^ (1)

The variables k and denote the parameters of the Mark–Houwink equation. The values of k and α are 3.73 × 10^−4^ and 0.66, respectively, in this work [30,31].

### 3.7. Long-Term Thermal Stability

Long-term thermal ability was tested in the glove box, which was pumped till vacuum, and high purity nitrogen was injected inside many times to ensure the oxygen concentration was lower than 10 ppm. The polymer solution was produced at a concentration of 0.5% and diluted to 0.175% in simulated Bohai saltwater. The diluted solution (2000 mL) was transferred and sealed in 10 ampoules with same size and aging container. After that, it was aged in a constant-temperature drying oven set to 75 °C. After a period, a 16 mL diluted solution was removed from the ampoule and the viscosity was measured and recorded. All of the treatment processes above were performed out in the glove box.

### 3.8. Core Flooding Test

At 75 °C, core flooding tests were conducted to determine the propagation of polymer solutions in a porous medium and the enhanced oil recovery efficiency. Table S4 summarizes the fundamental characteristics of the artificial cores (CNOOC EnerTech-Drilling and Production Co., Tianjin, China) utilized in the tests.

The injectivity test demonstrates a distinct flow behavior of polymer solution in porous media using three consecutive injection systems: brine, polymer solution, and following brine. For polymer flooding, a continuous injection rate of 1.0 mL/min was utilized. Finally, varied steady pressure reductions caused by injection systems, as determined by the resistance factor (RF) and residual resistance factor (RRF) in Equations (2) and (3) [32]:

RF = ΔP_p_/ΔP_wb_ (2)

RRF = ΔP_wa_/ΔP_wb_ (3)

where ΔP_wb_, ΔP_p_, and ΔP_wa_ denote the pressure differential between the first water process, the polymer flooding process, and the post-water flooding process.

The purpose of the oil displacement test was to see the contribution of polymer flooding to the improvement of recovery efficiency (for the comparison of water drive). The synthetic saline was injected into the rock core for 1.0 PV. Then the fixed slug of 0.3 PV polymer solution was injected to recycle the residual oil. It was used to pursue water drive at the same injection pressure until the production was increased again. The enhanced oil recovery was described as the volume ratio of accumulated oil production to the original saturation, and the recovery factor E_p_ can be calculated as follows [33,34]:

E_p_ = E_t_ − E_w_ (4)

where E_t_ is the total recovery factor during the oil displacement process, and E_w_ is the total recovery factor of the initial water flooding before polymer injection.

## 4. Conclusions

In this study, AM, AA, AMPS, and POM were used as comonomers to prepare a hydrophobically associating HAP polymer by free radical polymerization within the aqueous solution, starting at 0 °C. The purpose for preparing HAP is for use in polymer flooding in offshore oilfields, especially Bohai oilfields (temperature 75 °C; salinity, 3.26 × 10^5^ mg/L). The ^1^H NMR spectrum shows that the molecular structure of HAP is the same as expected. The viscosity-average molecular weight of HAP polymer obtained by the optimum reaction conditions was tested as 2.1 × 10^7^. The dissolution behavior and solution properties of the prepared HAP and commercial HPAM with similar molecular weight were studied systematically. Compared with HPAM, the HAP polymer had much higher solution viscosity. The dissolution times of HAP and HPAM polymers were 31 min and 14 min, respectively, in Bohai seawater. The viscosity retention of HAP was 64.6% after 60 days of aging at 75 °C, which was 13.9% higher than that of the HPAM solution, proving its excellent anti-ageing effect. The RF and RRF of HAP were higher than those of HPAM when both the concentration and the core permeability were the same in the application of the oil reservoir. Compared with HPAM, the HAP solution with a concentration of 0.175% had a much better oil drive effect in the porous medium, which can improve oil production by 8.8%.

## Figures and Tables

**Table 1:** The composition of simulated Bohai seawater, supplied by CNOOC EnerTech-Drilling and Production Co.

Cation			Anion				
Na^+^ (mg/L)	Ca^2+^ (mg/L)	Mg^2+^ (mg/L)	CO_3_^2−^ (mg/L)	HCO_3_^−^ (mg/L)	SO_4_^2−^ (mg/L)	Cl^−^ (mg/L)	Salinity (mg/L)
10,328.53	368.98	1187.65	12.96	145.98	2239.01	18,327.98	32,611.09

**Table 2:** The property comparison of HAP and HPAM.

Sample	Solid Content	Molecular Weight	Dissolution Time ^a^	Solution Viscosity ^a^
HAP	92%	2.1 × 10^7^	32 min	55.8 mPa⋅s
HPAM	91%	2.2 × 10^7^	14 min	26.3 mPa⋅s

^a^ Polymer concentration, 0.175%; salinity, 3.26 × 10^5^ mg/L; shear rate, 7.34 s^−1^.

## Data Availability

The data presented in this study are available in Supplementary Materials.

## Supplementary Materials

The following supporting information can be downloaded at: https://www.mdpi.com/article/10.3390/molecules27092744/s1, Figure S1: Effect of components and reaction conditions of polymerization under low temperature on dissolution time and viscosity of HAP polymer; Figure S2: Variation of pH with salinity in HAP, HPAMM solutions and simulated Bohai seawater as well as dilute solutions at 75 °C (Polymer concentration, 0.175%; shear rate, 7.34 s^−1^); Table S1: The influence of monomer and initiator dosage on dissolution time and solution viscosity; Table S2: The influence of reaction condition (the amount of cosolvent and chain transfer agent; pH value) on dissolution time and solution viscosity; Table S3: The effect of reaction time and temperature on dissolution time and solution viscosity; Table S4: Basic parameters of the artificial cores used in this work; Table S5: RF and RRF results for solutions of HAP and HPAM (Polymer concentration, 0.175%); Table S6: Summary of recovery factors for aqueous solutions of HAP and HPAM.
