# Supplementary material for: Hydrophobically Associating Polymers Dissolved in Seawater for Enhanced Oil Recovery of Bohai Offshore Oilfields
Source: Molecules. 2022 Apr 24;27(9):2744. doi: 10.3390/molecules27092744 (PMC9104856; doi:10.3390/molecules27092744)
Supplement: Supplementary file 1 [file molecules-27-02744-s001.zip › molecules-1688495-supplementary.pdf]

Supporting information for

Hydrophobically Associating Polymers Dissolved in Seawater for Enhanced Oil Recovery of Bohai Offshore Oilfields

Fei Yi [1,2], Bo Huang [1,3], Chengsheng Wang [1,2], Xiaoxu Tang [4], Xiujun Wang [1,3], Quangang Liu [1,2], Yanhui Su [1,2], Shijia Chen [1,2], Xiaoyan Wu [1,2], Bin Chen [1,2], Jing Zhang [1,2], Dianguo Wu [5], Shuai Yu [5], Yujun Feng [5] and Xin Su [5,*]

[1] State Key Laboratory of Offshore Oilfield Exploitation, Tianjin 300452, China;
[2] CNOOC EnerTech-Drilling and Production Co., Tianjin 300452, China
[3] CNOOC Research Institute, Beijing 100027, China
[4] Tianjin Branch of CNOOC Ltd., Tianjin 300451, China
[5] Polymer Research Institute, State Key Laboratory of Polymer Materials Engineering, Sichuan University, Chengdu 610065, China
* Correspondence: xinsu@scu.edu.cn

Table S1. The influence of monomer and initiator dosage on dissolution time and solution viscosity

No.	AM (g)	AA (g)	AMPS (g)	POM (g)	VA-044 (g)	$NaHSO_3$ (g)	$(NH_4)_2S_2O_8$ (g)	Hydrolysis Degree (%)	Dissolution Time[a] (min)	Solution Viscosity[a] (mPa·s)
**N1**	56	0	15	7	0.01	0.01	0.01	0	20	10.3
**N2**	184	35	15	7	0.01	0.01	0.01	13	30	21.5
**N3**	184	70	15	7	0.01	0.01	0.01	25	70	44.5
**N4**	184	70	30	7	0.01	0.01	0.01	26	65	47.3
**N5**	184	70	45	7	0.01	0.01	0.01	23	60	54.5
**N6**	184	70	60	7	0.01	0.01	0.01	25	60	55.2
**N7**	184	70	60	7	0.01	0.01	0.02	25	55	49.1
**N8**	184	70	60	7	0.01	0.01	0.03	25	60	47.5
**N9**	184	70	60	7	0.01	0.01	0.04	25	65	34.2
**N10**	184	70	60	7	0.01	0.01	0.05	26	65	23.2
**N11**	184	70	60	7	0.01	0.01	0.06	25	70	15.6
**N12**	184	70	60	7	0.01	0.01	0	25	65	10.3
**N13**	184	70	60	10	0.01	0.01	0.01	25	125	99.1
**N14**	184	70	60	9	0.01	0.01	0.01	25	105	89.6
**N15**	184	70	60	8	0.01	0.01	0.01	25	95	73.4
**N16**	184	70	60	6	0.01	0.01	0.01	25	55	37.6
**N17**	184	70	60	5	0.01	0.01	0.01	25	50	28.5
**N18**	184	70	60	4	0.01	0.01	0.01	25	40	21.6
**N19**	184	70	60	3	0.01	0.01	0.01	25	30	17.7
**N20**	184	70	60	2	0.01	0.01	0.01	25	25	9.8

[a] Polymer concentration, 0.175%; particle size of polymer powder, 40-120 mesh; salinity, 3.26×10^5 mg/L; shear rate, 7.34 s^{-1}

Table S2. The influence of reaction condition (the amount of cosolvent and chain transfer agent; pH value) on dissolution time and solution viscosity

No.	AM (g)	AA (g)	AMPS (g)	POM (g)	VA-044 (g)	$NaHSO_3$ (g)	$(NH_4)_2S_2O_8$ (g)	pH	Hydrolysis Degree (%)	Dissolution Time[a] (min)	Solution Viscosity[a] (mPa·s)
**N21**	184	70	60	7	0.015	0.01	0.01	8	24	50	57.3
**N22**	184	70	60	7	0.02	0.01	0.01	8	25	50	55.1
**N23**	184	70	60	7	0.025	0.01	0.01	8	25	45	52.1
**N24**	184	70	60	7	0.003	0.01	0.01	8	25	40	49.0
**N25**	184	70	60	7	0.035	0.01	0.01	8	24	35	46.3
**N26**	184	70	60	7	0.04	0.01	0.01	8	25	35	42.1
**N27**	184	70	60	7	0.045	0.01	0.01	8	23	30	39.2
**N28**	184	70	60	7	0.05	0.02	0.01	8	24	25	35.1
**N29**	184	70	60	7	0.01	0.03	0.01	8	25	55	56.2
**N30**	184	70	60	7	0.01	0.05	0.01	8	24	50	61.5
**N31**	184	70	60	7	0.01	0.06	0.01	8	25	45	58.7
**N32**	184	70	60	7	0.01	0.01	0.01	8	23	35	56.5
**N33**	184	70	60	7	0.01	0.02	0.01	8	24	35	54.2
**N34**	184	70	60	7	0.01	0.03	0.01	8	25	30	49.1
**N35**	184	70	60	7	0.01	0.10	0.01	8	25	25	41.0
**N36**	184	70	60	7	0.01	0.01	0.01	2	23	N/A[b]	N/A[b]
**N37**	184	70	60	7	0.01	0.01	0.01	4	23	N/A[b]	N/A[b]
**N38**	184	70	60	7	0.01	0.01	0.01	6	23	55	60.0
**N39**	184	70	60	7	0.01	0.01	0.01	8	23	40	56.5
**N40**	184	70	60	7	0.01	0.01	0.01	10	23	40	49.2
**N41**	184	70	60	7	0.01	0.01	0.01	12	23	35	37.1

[a] Polymer concentration, 0.175%; particle size of polymer powder, 40-120 mesh; salinity, 3.26×10^5 mg/L; shear rate, 7.34 s^{-1}

[b] The polymers do not dissolve, so their aqueous solution viscosity and dissolution time cannot be measured.

Table S3. The effect of reaction time and temperature on dissolution time and solution viscosity

No.	AM (g)	AA (g)	AMPS (g)	POM (g)	VA-044 (g)	$NaHSO_3$ (g)	$(NH_4)_2S_2O_8$ (g)	Reaction Time (h)	Hydrolysis Degree (%)	Reaction Temperature (°C)	Dissolution Time[a] (min)	Solution Viscosity[a] (mPa·s)
**N42**	184	70	60	7	0.01	0.01	0.01	3	24	0	10	6.4
**N43**	184	70	60	7	0.01	0.01	0.01	6	25	0	10	17.9
**N44**	184	70	60	7	0.01	0.01	0.01	9	25	0	25	36.1
**N45**	184	70	60	7	0.01	0.01	0.01	12	25	0	27	54.2
**N46**	184	70	60	7	0.01	0.01	0.01	15	24	0	32	55.6
**N47**	184	70	60	7	0.01	0.01	0.01	12	25	0	40	56.2
**N48**	184	70	60	7	0.01	0.01	0.01	12	23	10	35	46.1
**N49**	184	70	60	7	0.01	0.01	0.01	12	24	20	30	32.6
**N50**	184	70	60	7	0.01	0.01	0.01	12	25	30	30	19.5
**N51**	184	70	60	7	0.01	0.01	0.01	12	24	40	30	11.3
**N52**	184	70	60	7	0.01	0.01	0.01	12	25	0	30	55.2

[a] Polymer concentration, 0.175%; particle size of polymer powder, 40-120 mesh; salinity, 3.26×10^5 mg/L; shear rate, 7.34 s^{-1}

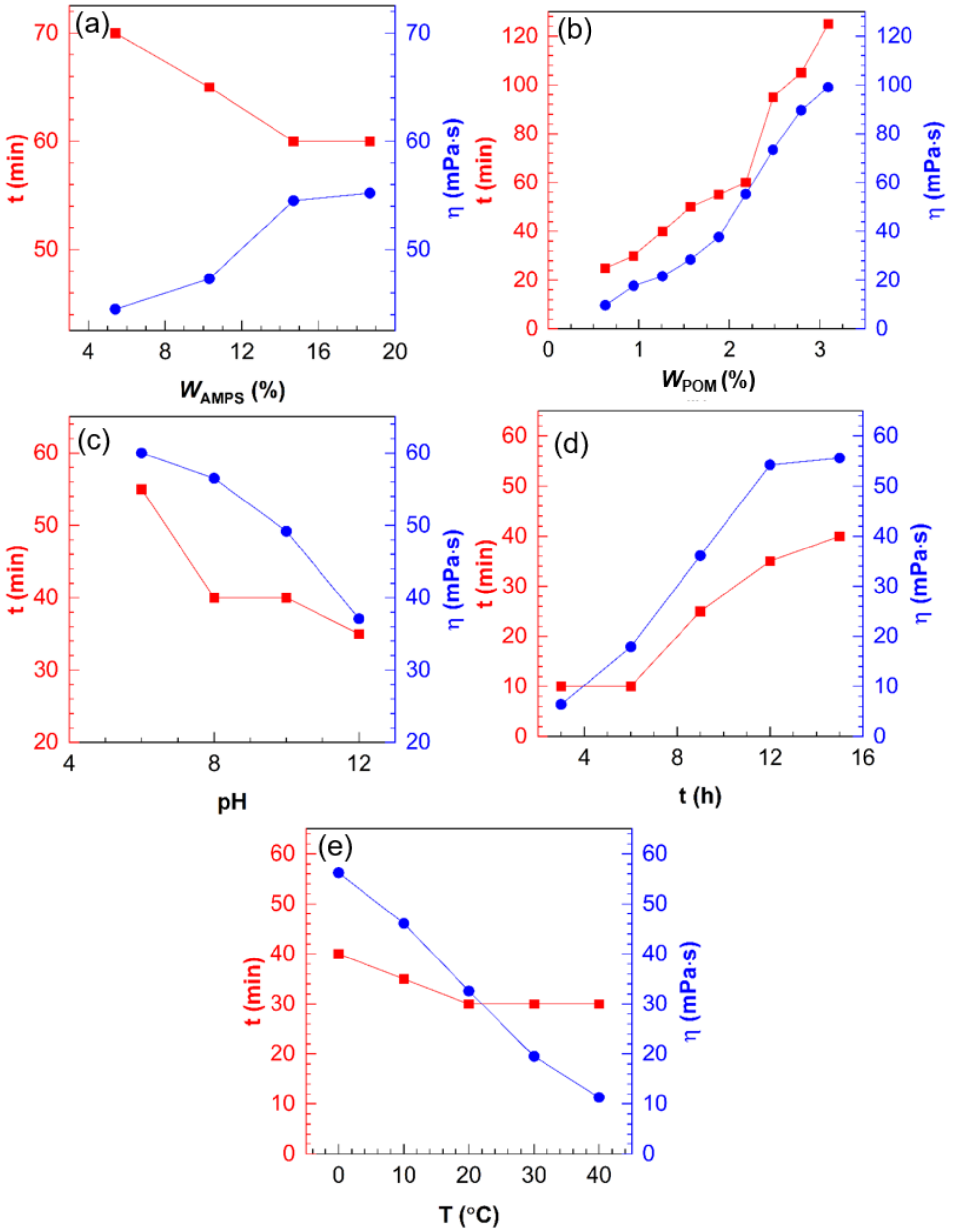

Figure S1. Effect of components and reaction conditions of polymerization under low temperature on dissolution time and viscosity of HAP polymer. (a) Content of AMPS comonomer and the data from N3-N6 in Table S1; (b) Content of POM comonomer and the data from N6, N13-N20 in Table S1; (c) pH value for solution of reactants prior to polymerization and the data from N36-N41 in Table S2; (d) reaction time of polymerization and the data from N42-N46 in Table S3; (e) Temperature of the reaction system at the beginning of polymerization the data from N48-N52 in Table S3.

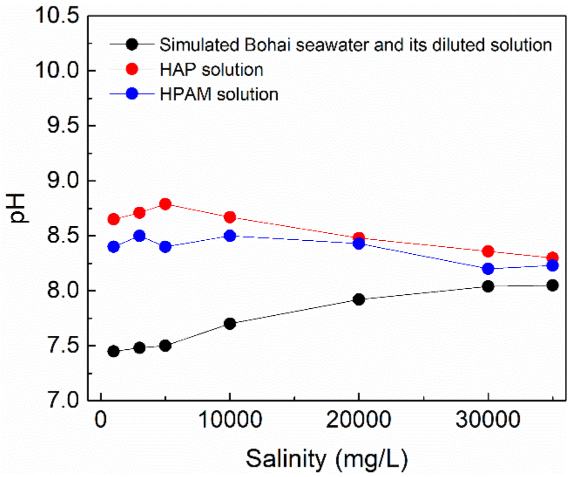

Figure S2. Variation of pH with salinity in HAP, HPAMM solutions and simulated Bohai seawater as well as dilute solutions at 75°C (Polymer concentration, 0.175%; shear rate, 7.34 s^{-1}).

Table S4. Basic parameters of the artificial cores used in this work.

Core	*L* (cm)	*d* (cm)	PV (mL)	ϕ (%)	K_w (mD)
No. 1	10.1	2.5	12.5	15.9	331
No. 2	10.0	2.5	12.4	16.1	333
No. 3	10.1	2.5	13.6	16.2	342
No. 4	10.1	2.5	13.2	16.0	339

Table S5. RF and RRF results for solutions of HAP and HPAM (Polymer concentration, 0.175%).

Core	Polymer sample	Permeability	RF	RRF
No. 1	HAP	240 mD	38.7	14.3
No. 2	HPAM	254 mD	25.3	9.5

Table S6. Summary of recovery factors for aqueous solutions of HAP and HPAM.

Core	Samples	E_w (%)	E_p (%)	E_T (%)
No. 3	HAP	22.4	19.7	42.1
No. 4	HPAM	22.5	10.9	33.4

E_w denotes the incremental oil recovery after initial water-flooding.

E_p refers to the incremental oil recovery after polymer injection.

E_T represents the total recovery.
